# Criminal victimization, cognitive social capital and mental health in an urban region in Germany: a path analysis

**DOI:** 10.1007/s00127-020-02021-5

**Published:** 2021-01-06

**Authors:** Reinhold Kilian, Annabel Müller-Stierlin, Natalie Lamp, Carolin von Gottberg, Thomas Becker

**Affiliations:** Department of Psychiatry II, Ulm University, Bezirkskrankenhaus Günzburg, Lindenallee 2, 89312 Günzburg, Germany

**Keywords:** Criminal victimization, Mental health, Depression, Anxiety, Cognitive social capital

## Abstract

**Purpose:**

There is ample evidence that experiencing a criminal victimization is associated with lasting emotional problems among victims. To date, the mechanisms behind this association are not well understood. Based on the theoretical assumptions derived from a transactional stress-appraisal and coping model this study analyses the role of cognitive social capital (SC) in the association between criminal victimization (CV) and victims’ mental health.

**Methods:**

A cross-sectional, computer-aided telephone survey including a representative sample of 3005 persons from three German cities was conducted. Respondents were asked about CV during their lifetime, cognitive SC, perceived victimization risk, perceived safety and perceived ability to prevent victimization. The PHQ-4 was used as a measure of anxiety and depression. The data were analyzed by means of logistic regression models and a path model controlled for sociodemographic characteristics.

**Results:**

Lifetime CV with any type of crime was associated with a clinically relevant increased risk of mental disorder (PHQ-4 ≥ 9; OR 1.8, *p* ≤ 0.05). Path analyses revealed that the direct association between CV and PHQ-4 (*β* = 0.454; *p* ≤ 0.01) was significantly diminished by cognitive SC (*β* =  − 0.373; *p* ≤ 0.05).

**Conclusion:**

Our results suggest that cognitive SC is an individual resilience factor against negative experiences related to CV and that it holds the potential to diminish negative mental health consequences of CV. Further research should explore to what extent an enhancement of cognitive SC can help to prevent anxiety and depression among crime victims.

**Supplementary Information:**

The online version contains supplementary material available at 10.1007/s00127-020-02021-5.

## Introduction

Beyond physical injury and property loss, the experience of being a victim of a criminal act has both short- and long-term negative effects on mental health [1–12]. Although the symptoms of posttraumatic stress disorder (PTSD) are the most severe psychological sequelae of violent offenses [3, 10, 12–16], several researchers have identified a broad spectrum of other psychological symptoms among victims of violent and nonviolent crime [2, 4, 6–10, 17, 18]. As Tan and Haining [8] showed, 86% of crime victims questioned in Sheffield (UK) reported at least one psychological symptom as a consequence of a crime experience: stress (59.7%), sleeping difficulties (39.3%), lack of confidence (37.2%), depression (30.1%) and panic attacks (24.5%). The results of longitudinal studies indicate that the negative psychological effects of crime victimization can last for more than 12 months [1, 2, 7] and that the use of mental health services is effective at reducing symptoms [1]. Results from a longitudinal twin study in the UK indicate that childhood crime victimization increases the risk of psychotic symptoms during adolescence [9]. Majority of studies are focused on violent crimes and therefore the knowledge about the effects of minor criminal offences is rare. Only recently, a study from the Netherlands revealed that victims of physical violence had a significantly higher risk of reporting PTSD symptoms than victims of burglary or fraud [19].

### Explanations for the negative effects of criminal victimization on mental health

From the perspective of the transactional-stress and coping model (TSCM), a criminal act is regarded as an environmental stimulus whose psychological effect depends on a cognitive appraisal of a person’s adaptation requirements (primary appraisal), coping resources (secondary appraisal) and the individual’s ability to meet the adaptation requirements and acquire the coping resources (reappraisal) [4, 10, 20]. Negative psychological consequences are expected when individuals appraise their coping resources not sufficient to adapt to an environmental stimulus in a way that prevents negative consequences. In the case of a criminal victimization, the stimulus is not only defined by the threat of physical injury or property loss but also by the violation of basic human assumptions about the benevolence of other people and the safety of the living environment [10]. Therefore, most people can be expected to appraise the stimulus of a criminal victimization as an elementary threat that induces secondary appraisal of the capacity to avoid physical injury or property loss [10]. A prior experience of not being able to prevent the negative consequences of a criminal victimization may therefore evoke feelings of low self-efficacy and helplessness (which may also be generalized to similar future situations) [10]. Such generalized expectations of helplessness and low self-efficacy are major sources of depression [5, 21]. Therefore, after an attack, not only the victim's appraisal of the original events but also their expectations regarding future similar events affect the psychological consequences they experience [10]. Environmental and individual factors are expected to influence the outcome of this appraisal process.

In past studies, crime rates and signs of neighborhood incivility have increased perceived victimization risk and fear of crime [22–24] but have not directly affected the psychological consequences of criminal victimization [22, 25].

From the perspective of trauma psychology, social support is considered a crucial factor in preventing the negative consequences of criminal victimization on mental health by strengthening the victims’ trust in the benignity and helpfulness of other people [10]. This hypothesis has been partly supported by results of the Sheffield case study, indicating that crime victims who lived alone reported stronger negative effects than those who lived with someone else [8]. In addition, Haden and Scarpa [4] revealed that perceived social support diminished victimization’s effect on depressive symptoms.

### The role of social capital in explaining the aftermath effects of criminal victimization on mental health

The concept of social capital (SC) has been introduced by Bourdieu [26] to emphasize the roles that social relationships play in the central dimensions of social distinction and social inequality in modern societies. Coleman [27] integrated the sociological and economic theories of human action so as to define SC as an individual and collective resource analogous to financial and human capital. In his view [27], social capital (SC) is generated by the individuals’ willingness to invest in mutual trust, information exchange and an orientation toward social norms; this investment results in both individual and collective benefits [27]. Putnam [28] used the term SC, in its most popular form, to stress the roles that civic participation and the norm of general reciprocity play in modern democracies. Despite its widespread use in fields of political economy and public health, a general definition of SC is still lacking and it is criticized for conceptual ambiguity and imprecise application [29–31]. To summarize the different dimensions of SC referred to in the literature, Islam et al. [32] differentiate between structural and cognitive SC. While structural SC refers to objective aspects of social organization, such as the density of social networks, or patterns of civic engagement, cognitive SC refers to subjective representations of the level of interpersonal trust, sharing and reciprocity [32]. Structural and cognitive SC have been found to be positively associated with both physical health [32–36] and mental health [37–40]. Nevertheless, there is no single theory of the mechanisms by which social capital operates, nor of whether it operates at the individual or the social organization level or both [30, 32, 36]. Moreover, some authors have argued that the SC concept as it is applied in public health may obscure the health related effects of social structures and material living conditions underlying the objective and subjective characteristics of social relationships [31, 32]. With regard to mental health outcomes, the results of several systematic reviews indicate homogenous inverse associations between mental health and individual level cognitive SC but only weak or no associations of mental health with structural SC [39, 41, 42]. These results might reflect the fact that most of the studies included used measures of cognitive SC only but they also support the position of Portes [30] that SC is only a new “more appealing” term for well-established individual level characteristics such as social support or social contacts [30]. In sum, both the current state of theoretical debates and the empirical evidence support the assumption that cognitive SC defined as people’s subjective representation of the quality of social relationships in their life-worlds can affect their ability to cope with psychological challenges such as criminal victimization.

### How could cognitive social capital moderate the association between criminal victimization and mental health?

According to the distinction between structural and cognitive SC provided by Islam et al. [32] we expect cognitive SC defined as the individual perception of interpersonal trust, mutual neighborhood support and reciprocity of social relationships to affect each of the three steps of the appraisal process presumed by the TSCM [20]. In the primary appraisal, individuals’ trust in the benevolence of other people and reciprocity of social relationships may reduce their perceived risk of future victimization [23, 43–46]. In secondary appraisal, a general trust in the helpfulness of people may strengthen an individual’s sense of self-efficacy in terms of their defense against potential losses and injuries caused by a criminal victimization [20, 46]. As a moderating variable in this appraisal process, we would expect that a higher level of cognitive SC might be associated with a lower vulnerability of crime victims against the negative psychological consequences of a criminal victimization.

However, considering the assumptions of trauma psychology [10], a victim’s belief in the helpfulness and the benevolence of other people might also be weakened by the experience of a criminal victimization. In this case, the negative psychological effects of criminal victimization would be increased by the deterioration of cognitive SC and in turn by an increase of the perceived vulnerability to future victimization and its negative consequences.

### Study aims and hypotheses

The aim of this analysis is to investigate the bearing that cognitive SC has on the association between criminal victimization and mental health, in the framework of the theories discussed above. For this purpose, we developed a path model including the core variables in line with the theoretical perspectives discussed above:

If cognitive SC works as a buffer against the negative effects of criminal victimization on mental health we would expect moderating associations between cognitive SC and the paths from criminal victimization to perceived future victimization, perceived safety and on mental health. If, on the contrary, cognitive SC will be shattered by the experience o of criminal victimization we would expect criminal victimization to have a direct negative effect on cognitive SC, in turn indirectly leading to increased vulnerability and decreased mental health.

## Materials and methods

### Study design and sample

In this study, we utilized a cross-sectional, computer-assisted telephone survey of people 18 years and older living in three adjacent German cities with a total population of 1.4 million, from November 2015 through January 2016. USUMA Ltd., a social and marketing research company located in Berlin, conducted the survey. For the purpose of limiting the survey to the population of three selected cities, sample selection was restricted to households with landline phone numbers which include a regional code. At the time of the survey about 91% of all households in Germany still had a landline phone number [47], and the sampling strategy is therefore not regarded as limiting the representativeness of the sample. Phone numbers were randomly drawn from the phone registry and supplemented with unregistered numbers by means of the Gabler–Häder method [48]. Household members were selected using the Kish [49] selection grid. The research company contacted 6780 households. To ensure that these households were located in the study region, the respondents confirmed their places of residence before the start of the interview. After excluding 69 households for being outside the study region, 3005 people (44.32% response rate) participated in the survey.

### Measures

We assessed criminal victimization using the following questions: “Have you been the victim of a burglary in your home? Have you been injured in a physical attack?” “Have you been the victim of a sexual assault?” The response categories were “never”; “yes, in the last year”; and “yes, more than a year ago”.

We assessed subjective risk of criminal victimization using this question: “How do you estimate the risk of being affected by any of the following events within the next 12 months?” The items related to being the victim of one (or several) of the following events: a terrorist attack, a home burglary, a physical attack, and a sexual assault. The response categories were “very low” (1), “low” (2), “moderate” (3), “high” (4) and “very high” (5).

We assessed perceived personal safety using the following three questions: “How safe do you feel in your daily life?” “Do you consider the area where you live to be safe?” “Are you worried about your personal safety?” The response categories were “not at all” (1), “a little” (2), “moderate” (3), “quite” (4) and “extremely” (5).

We assessed perceived ability to prevent future criminal victimization using the following questions: “How do you rate your ability to protect yourself against a burglary in your home?” “How do you rate your ability to protect yourself against a physical attack?” “How do you rate your ability to protect yourself against a sexual assault?” The response categories were “very low” (1), “low” (2), “moderate” (3), “high” (4) and “very high” (5).

Given the lack of a unique measurement construct of SC [50–53] we selected those dimensions discussed in the literature [32] which seemed to be most relevant for our research question.

In the absence of a uniform instrument to measure the different dimensions of social capital [52] we measured cognitive social capital according to our working definition as the perceived quality of neighborhood contacts, the perceived level of mutual neighborhood support and the perceived reciprocity norm using questions from the German General Social Survey [54].The quality of neighborhood contacts was assessed by the question: “How would you rate the contacts in your neighborhood?” This had the following response categories: “Almost nobody knows or greets each other here”, “People greet each other but have little contact with each other”, “Most people know and help each other”, and “People have close contacts and shared leisure activities”. The level of mutual neighborhood support was assessed using the question “Who helps whom in your neighborhood?” For the items “I help others” and “Others help me,” the answer categories were “yes” (1) and “no” (0); for the items “I do not help anyone” and “Nobody helps me,” the answer categories were “yes” (1) and “no” (0). Another question (“Do you leave a key with a neighbor when you are away for a long time?”) had the answer categories “no” (0) and “yes” (1). Reciprocity expectations, using the level of agreement with the following statements: “I am convinced that, in Germany, in an emergency situation, everyone gets the help they need.”; “In an emergency situation, I would also offer my help to unknown people”; “In an emergency situation, I can expect help from people I do not know”; and “You can trust most people.” The response categories were “not true at all” (1), “mostly untrue” (2), “partially true” (3), “mostly true” (4) and “completely true” (5).

We assessed symptoms of anxiety and depression using the brief version of the Patient Health Questionnaire (PHQ-4) [55]. The PHQ-4 is a self-report instrument consisting of a 2-item depression scale (PHQ-2) for assessing loss of interest and depressed mood and a 2-item anxiety scale (GAD-2) for assessing nervousness and worry. The PHQ-4 has good psychometric properties (Cronbach’s alpha of 0.78), as do its two subscales PHQ-2 (Cronbach’s alpha = 0.75) and GAD-2 (Cronbach’s alpha = 0.82), and these scales are suitable for both population surveys and clinical use [55]. PHQ-4 scores of 6 (indicating a yellow flag) or 9 (indicating a red flag) are the recommended clinical cutoff values to indicate the presence of general anxiety or depressive disorder. For the PHQ-2 and GAD-2 subscales, values of 3 (yellow flag) or 5 (red flag) are the recommended clinical cutoff values for the presence of depression or general anxiety disorder [55].

### Control variables

Because of associations with criminal victimization or any other of our model variables reported in the literature discussed in the introduction we included the following control variables in our analyses: age (in years), gender (male = 0; female = 1), education (below Abitur, i.e. below the level qualifying for university = 0; Abitur and above = 1); income (monthly net household income categorized from 1 = 1.000 € to 9 = 4.500 € and above), unemployment (0 = no; 1 = yes), partnership status (0 = living alone; 1 = living with a partner)..

### Statistical analyses

We used sample weights provided with the data set in all analyses to correct for sampling bias with regard to household size, gender, age and education (Gabler, Hoffmeyer-Zlotnik, and Krebs, 1994) [55].

We fitted logistic regression models estimating the associations between being the victim of home burglary, physical assault, sexual assault or of any type of these crimes over the lifetime, on the cutoff values suggested by Löwe et al. [55] for the PHQ-2 (PHQ-2 ≥ 3), the GAD-2 (GAD-2 ≥ 3) and the PHQ-4 (PHQ-4 yellow flag ≥ 6 and red flag ≥ 9) as dependent variables, adjusted for the control variables mentioned above.

For further analyses, we built an index of the lifetime experience of criminal victimization with the categories: 0 = no victimization, 1 = one type of criminal victimization, 2 = two types of criminal victimization, 3 = three types of criminal victimization.

**Fig. 1:**
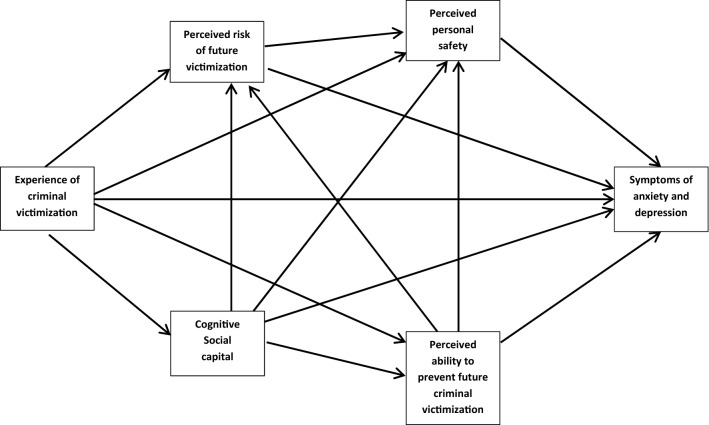
Theoretical model of the role of social capital in the associations between criminal victimization and mental health

We computed a structural equation model consisting of six regression equations (see electronic appendix), using the components of our theoretical model presented in Fig. 1 as dependent variables. In the applied SEM these equations will be estimated simultaneously by means of a maximum likelihood approach with robust standard errors (MLR) to take into account non normal distribution of dependent variables [56]. Each regression equation includes the variables defined by the paths of our theoretical model presented in Fig. 1 and a common set of control variables described above. This makes sure that all presented regression parameters are adjusted for the same set of control variables. Beyond estimating the parameters of the statistical associations (path coefficients) the SEM approach tests the hypothesis that the covariance structure of the included variables is sufficiently represented by the defined model paths. To account for moderating associations we included the product term of CV and cognitive SC in each equation. As a result of our model specification, each path coefficient has been statistically adjusted for the same set of control variables.

We tested the model’s fit by means of the root-mean-square error of approximation (RMSEA), which had to be less than 0.05, the comparative fit index (CFI) and Tucker–Lewis index (TLI), each of which had to be greater than 0.90 [57]. We calculated the indirect variance components by decomposing the total variance components [58]. Path analyses were conducted with MPLUS 7 [56]. For computing the logistic regression models and Cronbach’s alpha we used STATA 15 [59].

## Results

### Sample characteristics

The characteristics of the study sample are presented in Table 1. The survey sample comprises 3005 people. The comparison of the original and weighted characteristics indicates that various groups—older people, women, those with high education levels, and those who had a monthly household income of 3000 € and above—were overrepresented in the original sample.

**Table 1:** Original and weighted sample characteristics

	Sample (*n* = 3005)	Weighted sample^a^ (*n* = 3005)
Female, *n* (%)	1601 (53.3)	1556 (51.8)
Age, M (SD)	53.5 (18.7)	50.24 (18.8)
Higher education, *n* (%)	1536 (51.1)	911 (30.3)
Unemployed, *n* (%)	76 (2.53)	117 (3.9)
Living with partner, *n* (%)	1367 (45.5)	1530 (50.9)
Monthly household income ≥ 3000 €, *n* (%)	1097 (36.5)	952 (31.7)

^a^Weighted at the city or district level for age, gender and education

### Prevalence of CV

As shown in Table 2, 899 (30.0%) study participants had been victims of a crime at least once in their lives. During the 12 months prior to the survey, 177 (5.9%) people had experienced at least one criminal act. The most commonly experienced crime was burglary, with a lifetime prevalence of 21.1% and a 12-month prevalence of 4.4%. Although the prevalence of burglary did not differ between men and women, the prevalence for physical injury was twice as high in men compared to women, and the prevalence of sexual assault among women was 3.5 times that among men.

**Table 2:** Lifetime and 12 month prevalence of criminal victimization (*n* = 3005)

	Male	Female	Total
Burglary ever, *n* (%)	293 (20.9)	341 (21.3)	634 (21.1)
Burglary last year, *n* (%)	66 (4.7)	66 (4.1)	132 (4.4)
Physical assault ever, *n* (%)	205 (14.6)	97 (6.1)	306 (10.2)
Physical assault last year, *n* (%)	26 (1.9)	15 (1.0)	42 (1.4)
Sexual assault ever, *n* (%)	22 (1.6)	71 (4.4)	91 (3.4)
Sexual assault last year, *n* (%)	4 (0.3)	8 (0.5)	12 (0.4)
Any victimization ever, *n* (%)	456 (32.4)	441 (27.6)	899 (30.0)
Any victimization last year (%)	91 (6.5)	86 (5.4)	177 (5.9)

### Associations between CV and mental health

As shown in Table 3, lifetime victimization by any of the criminal offences investigated in our study is associated with an increased risk of reporting a clinically relevant level of anxiety symptoms according to the GAD-2 cut-off and with an increased risk of reporting depressive symptoms above the yellow flag cut-off. The experience of a sexual assault is also associated with an increased risk of reporting depressive symptoms above the red flag cut-off value of the PHQ-4. Compared to non-victims, participants who had experienced sexual assault in their lives had twice the risk of depression (for cutoff point PHQ-2 ≥ 3) and a 2.3 times the risk of anxiety disorders (for the cutoff point of GAD-2 ≥ 3). The yellow-flag cutoff point of the PHQ-4 (≥ 6) indicated a 2.0 risk factor, and the red-flag cutoff point (≥ 9) indicated a 2.7 risk factor. People who had been injured in a physical attack had 2.64 times the risk of exceeding the GAD-2 cutoff point for an anxiety disorder and 2.35 times the normal risk of exceeding the PHQ-4 yellow-flag cut-off point. A victimization experience for one of the three types of crime was also associated with an increased risk of exceeding all clinical cut-off points of anxiety or depressive disorder.

**Table 3:** Associations between criminal victimization and mental health with regard to clinical GAD-2, PHQ-2 and PHQ-4 cut-off values

	GAD-2 ≥ 3			PHQ-2 ≥ 3			PHQ-4 ≥ 6			PHQ-4 ≥ 9		
	OR^a^	95% CI		OR^a^	95% CI		OR^a^	95% CI		OR^a^	95% CI	
Burglary	1.37*	1.01	1.85	1.15	0.91	1.45	1.54**	1.16	2.05	1.58	0.99	2.50
Physical injury	2.64***	1.86	3.75	1.74***	1.30	2.32	2.35***	1.67	3.32	1.51	0.81	2.83
Sexual assault	2.30**	1.36	3.87	1.97**	1.27	3.05	1.96**	1.16	3.32	2.68**	1.28	5.65
Any of the above	1.68***	1.27	2.21	1.30*	1.06	1.61	1.67***	1.28	2.17	1.67*	1.08	2.58

^*^p ≤ 0.05; **p ≤ 0.01; ***p ≤ 0.001

^a^Adjusted for sex, age, education, income, partnership, unemployment

## Results of the path analysis

**Fig. 2:**
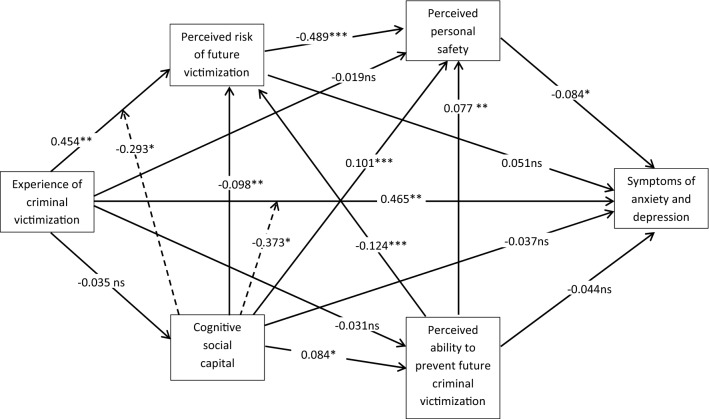
Standardized path coefficients of the associations between criminal victimization and mental health. [Solid lines represent direct associations, dotted lines represent coefficients of significant multiplicative terms (CV*SC)]. All parameters are adjusted for the following set of control variables: age (in years), gender (male = 0; female = 1), education (below the level qualifying for university = 0; level qualifying for university and above = 1); income (monthly net household income categorized from 1 = 1.000 € to 9 = 4.500 € and above), unemployment (0 = no; 1 = yes), partnership status (0 = living alone; 1 = living with a partner). *p < 0.05; **p < 0.01; ***p < 0.001; *ns* not significant

Standardized path coefficients (beta) are presented in Fig. 2. Path coefficients indicate a significant direct positive path from criminal victimization to symptoms of anxiety and depression (*β* = 0.465; se = 0.175; *p* = 0.008) but no significant paths to cognitive SC (*β* = -0.035; se = 0.023; *p* = 0.129), to respondents’ perceived ability to prevent future CV (*β* =  − 0.031; se = 0.023; *p* = 0.178), or to perceived safety (*β* =  − 0.019; se = 0.123; *p* = 0.875). While perceived personal safety was significantly negatively related to symptoms of anxiety and depression (*β* =  − 0.084; se = 0.039; *p* = 0.030), neither the path from social capital (*β* =  − 0.034; se = 0.030; *p* = 0.244) nor the path from perceived prevention ability (*β* =  − 0.044; se = 0.027; *p* = 0.107) to symptoms of anxiety and depression were significant.

Positive path coefficients from CV to the perceived risk of future victimization (*β* = 0.454; se = 0.147; *p* = 0.002) and negative path coefficients from cognitive SC to perceived risk (*β* =  − 0.098; se = 0.29; *p* = 0.001), and from perceived risk to perceived safety (*β* =  − 0.489; se = 0.023; *p* = 0.000) were significant while the path from perceived risk to symptoms of anxiety and depression was not (*β* = 0.051; se = 0.038; *p* = 0.185).

Significant path coefficients indicate positive associations between cognitive SC and perceived ability to prevent future CV (*β* = 0.084; se = 0.031; *p* = 0.006) and perceived safety (*β* = 0.101; se = 0.027; *p* = 0.000), between prevention ability and perceived safety (*β* = 0.077; se = 0.025; *p* = 0.002) and a negative association between prevention ability and perceived risk of future victimization (*β* =  − 0.124; se = 0.031; *p* = 0.000).

### Moderating associations

Path coefficients indicate significant negative interactions of cognitive SC on the association between CV and perceived risk of future CV (*β* =  − 0.293; se = 0.144; *p* = 0.042) and the association between CV of symptoms and anxiety and depression (*β* =  − 0.373; se = 0.164; *p* = 0.023) while the interaction effect of cognitive SC on the association between CV and perceived safety was not significant (*β* =  − 0.050; se = 0.122; *p* = 0.680).

### Indirect variance components

The deconstruction of the total variance components revealed a rather small significant total indirect variance component between CV and symptoms of anxiety and depression (*β* = 0.047; se = 0.023; *p* = 0.037) but no significant indirect variance component could be detected for particular combinations of model variables. We identified an indirect negative association between CV and perceived safety via the perceived risk of future CV (*β* =  − 0.232; se = 0.072; *p* = 0.001) and a positive indirect association between cognitive SC and perceived safety via the perceived risk of future victimization (*β* = 0.048; se = 0.014; *p* = 0.001).

### Model fit

As indicated by the *R*^2^ parameters, the model variables accounted for 0.01% of the variance in CV, 3.0% of the variance in cognitive SC, 11% of the variance in perceived risk of victimization, 11% of the variance in perceived ability to prevent future victimization, 31% of the variance in perceived safety, and 12% of the variance in symptoms of anxiety and depression.

The RMSEA of 0.035 (90% CI = 0.023–0.048; prob. RMSEA < 0.05 = 0.967), the CFI of 0.995, the TLI of 0.957 and the SRMR of 0.019 all indicate that the model fits the empirical covariance structure very well.

## Discussion

To our knowledge, this is the first study that empirically examines cognitive SC's role with regard to the association between CV and mental health, using a large representative sample from Germany. In accordance with prior studies from the US and UK, our results confirm that experiences of CV are associated with increased symptoms of anxiety and depression [1–8, 10]. As hypothesized in psychological trauma theory [10], experiences of sexual assault or physical attacks were associated with clinically relevant increases in the risk of suffering from a mental disorder. However, while the direct association between CV and mental illness symptoms identified in the path model is in accordance with trauma psychology [60] the lack of significant associations between cognitive SC and perceived safety fails to support the assumption that CV affects mental health indirectly through violating basic human assumptions about the safety of the world and the general benevolence and supportiveness of fellow humans[10].

Our results are in accordance with previous studies, indicating that the experience of a CV is associated with an increased perception of future victimization risk and a decreased feeling of personal safety. In addition, the association between CV and perceived safety is conditional to the perceived risk of future victimization, indicated by a significant strong indirect association and the absence of a significant direct association between CV and perceived safety. However, the association between personal safety and symptoms of mental disorder is significant but weak. As a consequence, only a small significant indirect association between CV and symptoms of mental disorder could be identified. While in line with the core assumptions of our model, these results provide no explanation of the association between CV and symptoms of mental disorder. On the contrary, the strong moderating association between cognitive SC mitigating the association between CV and symptoms of anxiety and depression and the perceived risk of future victimization is consistent with the hypothesis that general trust in the benevolence and helpfulness of other people has the potential to diminish the adverse consequences of negative life events such as CV on mental health [10, 37, 45]. This interpretation is in accordance with the results of previous studies [39, 61–64] in which lower levels of perceived SC were associated with higher risks or with more symptoms of mental illness, independent of the type of environmental demands.

Our results are in accordance with the assumptions of the transactional stress and coping theory which indicate that cognitive SC represents personal beliefs about the social environment that are directly associated with the subjective perception of a person’s ability to prevent future CV (which, in turn, is associated with an increased feeling of personal safety [20]). However, the lack of direct or indirect associations between cognitive SC and mental health does not corroborate the hypothesis that cognitive SC affects mental health by its association with increased perceived coping resources and personal safety [65].

In sum, these results are consistent with the general hypothesis that cognitive SC is an important individual resource among crime victims in maintaining mental health stable. The results of this study also reveal that the positive impact of cognitive SC cannot be interpreted by its effects on individual coping capacity, risk assessment or personal safety [66, 67] but is based mainly on its effect as a buffer against the negative impact of CV on mental health [68]. Moreover, cognitive SC not being negatively affected by the experience of CV suggests that it might be a stable component of individual belief systems that are not vulnerable to negative experiences [10]. To what extent, then can cognitive SC be enhanced by social or political interventions? In a recent systematic review Flores et al. [69] identified seven controlled quasi-experimental studies evaluating the effects of measures designed to enhance SC on mental health outcomes. Out of seven studies four found positive effects on individual level social capital and mental health [69]. Nonetheless, the results of these studies are of limited comparability due to differences in study methods and socio-cultural context. The authors conclude that in spite of promising results of a small number of studies more research is needed to clarify the effects of interventions aimed at improving mental health by enhancing SC [69].

## Limitations

The cross-sectional study design limits the interpretation of our findings with regard to the direction of causality. People with mental disorders present with an increased risk of CV [70], so there is a possibility that the statistical associations in our data set indicate that CV is a consequence of poor mental health, rather than vice versa. The study’s generalizability is limited because its sample is restricted to the inhabitants of three cities in the Ruhr region in Germany.

## Conclusions

Our results suggest that cognitive SC is an individual coping resource which is relatively inert to negative life experiences and has the potential to diminish negative consequences of CV on mental health. Further research should explore to what extent enhancement of cognitive SC at the individual or community level can help reduce anxiety and depression among crime victims.

## Supplementary Information

Below is the link to the electronic supplementary material.

Supplementary file1 (DOCX 27 KB)

Supplementary file2 (TXT 52 KB)

## Data Availability

Data of this investigation are available in the electronic supplement. Data file in fixed ASCII format: datasetsppe.txt. Data definitions: datasetsppedef.txt.
