# Supplementary material for: Criminal victimization, cognitive social capital and mental health in an urban region in Germany: a path analysis
Source: Soc Psychiatry Psychiatr Epidemiol. 2021 Jan 6;56(9):1565–74. doi: 10.1007/s00127-020-02021-5 (PMC8429151; doi:10.1007/s00127-020-02021-5)
Supplement: Supplementary file 1 — Supplementary file1 (DOCX 27 KB) [file 127_2020_2021_MOESM1_ESM.docx]

**Appendix 1: Regression equations, standardized path coefficients, standard errors, T values, p values and R^2^ of the structural equation model (SEM) presented in figure 2.**

**SEM Equation 1:**

Criminal victimization (CV) = b_0_Intercept + b_1_x_female_ + b_2_x_age_ + b_3_x_educ_ + b4x_income_ + b_5_x_unemployed_ + b_6_x_partner_

| **Tab Ap1: Standardized path coefficients of SEM equation 1 (R^2^ 0.001)** | | | | |
| --- | --- | --- | --- | --- |
|  | beta | s.e. | T | p |
| Female | -0.003 | 0.004 | -0.600 | 0.548 |
| Age | -0.006 | 0.005 | -1.247 | 0.212 |
| Education | -0.004 | 0.005 | -0.740 | 0.459 |
| Income | -0.014 | 0.006 | -2.287 | 0.022 |
| Unemployed | 0.010 | 0.008 | 1.139 | 0.255 |
| Partner | -0.010 | 0.005 | -2.092 | 0.036 |
| Intercept | 0.0661 | 0.028 | 23.379 | 0.000 |

**SEM equation 2:**

Perceived risk of future victimization = b_0_Intercept + b_1_x_CV_ + b_2_x_perceived ability_ + b_3_x_SC_ + b_4_x_CV*SC_ + b_5_x_female_ + b_6_x_age_ + b_7_x_educ_ + b_8_x_income_ + b_9_x_unemployed_ + b_10_x_partner_

| **Tab Ap2: Standardized path coefficients of SEM equation 2 (R^2^ = 0.110)** | | | | |
| --- | --- | --- | --- | --- |
|  | beta | s.e. | T | p |
| Criminal victimization (CV) | 0.454 | 0.147 | 3.096 | 0.002 |
| Perceived ability to prevent future CV | -0.124 | 0.031 | -3.983 | 0.000 |
| Cognitive social capital (SC) | -0.098 | 0.029 | -3.400 | 0.001 |
| Interaction term CV x SC | -0.293 | 0.144 | -2.028 | 0.043 |
| Female | 0.125 | 0.025 | 4.977 | 0.000 |
| Age | -0.126 | 0.028 | -4.469 | 0.000 |
| Education | -0.061 | 0.024 | -2.495 | 0.013 |
| Income | -0.076 | 0.030 | -2.564 | 0.010 |
| Unemployed | 0.009 | 0.027 | 0.320 | 0.749 |
| Partner | 0.127 | 0.027 | 4.773 | 0.000 |
| Intercept | 3.481 | 0.207 | 16.779 | 0.000 |

**SEM equation 3:**

Cognitive social capital (SC) = b_0_Intercept + b_1_x_CV_ + b_2_x_female_ + b_3_x_age_ + b_4_x_educ_ + b_5_x_income_ + b_6_x_unemployed_ + b_7_x_partner_

| **Tab Ap3:**  **Standardized path coefficients of SEM equation 3 (R^2^ = 0.030)** | | | | |
| --- | --- | --- | --- | --- |
|  | beta | s.e. | T | p |
| Criminal victimization (CV) | -0.035 | 0.023 | -1.520 | 0.129 |
| Female | 0.042 | 0.024 | 1.711 | 0.087 |
| Age | 0.015 | 0.028 | 0.537 | 0.591 |
| Education | 0.065 | 0.026 | 2.459 | 0.014 |
| Income | 0.071 | 0.029 | 2.409 | 0.016 |
| Unemployed | -0.075 | 0.035 | -2.135 | 0.033 |
| Partner | 0.063 | 0.026 | 2.437 | 0.015 |
| Intercept | 5.607 | 0.166 | 33.846 | 0.000 |

**SEM equation 4:**

Perceived ability to prevent future criminal victimization = b_0_Intercept + b_1_x_CV_ + b_2_x_SC_ + b_3_x_CV*SC_ + b_4_x_female_ + b_5_x_age_ + b_6_x_educ_ + b_7_x_income_ + b_8_x_unemployed_ + b_9_x_partner_

| **Tab Ap4: Standardized path coefficients of SEM equation 4 (R^2^ = 0.110)** | | | | |
| --- | --- | --- | --- | --- |
|  | beta | s.e. | T | p |
| Criminal victimization (CV) | 0.169 | 0.155 | 1.093 | 0.275 |
| Cognitive social capital (SC) | 0.084 | 0.031 | 2.730 | 0.006 |
| Interaction term CV x SC | -0.204 | 0.151 | -1.349 | 0.177 |
| Female | -0.111 | 0.025 | -4.340 | 0.000 |
| Age | -0.271 | 0.026 | -10.422 | 0.000 |
| Education | -0.043 | 0.024 | -1.791 | 0.073 |
| Income | 0.078 | 0.028 | 2.804 | 0.005 |
| Unemployed | -0.016 | 0.040 | -0.404 | 0.686 |
| Partner | -0.045 | 0.027 | -1.662 | 0.097 |
| Intercept | 3.481 | 0.207 | 16.779 | 0.000 |

**SEM equation 5:**

Perceived personal safety = b_0_Intercept + b_1_x_CV_ + b_2_x_perceived risk of future victimization_ + b_3_x_perceived ability_ + b_4_x_SC_ + b_5_x_CV*SC_ + b_6_x_female_ + b_7_x_age_ + b_8_x_educ_ + b_9_x_income_ + b_10_x_unemployed_ + b_11_x_partner_

| **Tab Ap5: Standardized path coefficients of SEM equation 5 (R^2^ = 0.311)** | | | | |
| --- | --- | --- | --- | --- |
|  | beta | s.e. | T | p |
| Criminal victimization (CV) | -0.019 | 0.123 | -0.158 | 0.875 |
| Perceived risk of a future criminal victimization | -0.489 | 0.023 | -21.310 | 0.000 |
| Perceived ability to prevent future CV | 0.077 | 0.025 | 3.696 | 0.000 |
| Cognitive social capital (SC) | 0.101 | 0.027 | 3.696 | 0.000 |
| Interaction term CV x SC | -0-050 | 0.122 | -0.412 | 0.680 |
| Female | 0.041 | 0.022 | 1.836 | 0.066 |
| Age | 0.002 | 0.026 | 0.082 | 0.935 |
| Education | 0.027 | 0.024 | 1.110 | 0.267 |
| Income | 0.060 | 0.031 | 1.956 | 0.050 |
| Unemployed | 0.016 | 0.027 | 0.584 | 0.559 |
| Partner | -0.053 | 0.023 | -2.320 | 0.020 |
| Intercept | 5.622 | 0.269 | 20.892 | 0.000 |

**SEM equation 6:**

Symptoms of anxiety and depression (PHQ 4) = b_0_Intercept + b_1_x_CV_ + b_2_x_perceived risk of future victimization_ + b_3_x_perceived ability_ + b_4_x _Perceived personal safety_ + b_5_x_SC_ + b_6_x_CV*SC_ + b_7_x_female_ + b_8_x_age_ + b_9_x_educ_ + b_10_x_income_ + b_11_x_unemployed_ + b_12_x_partner_

| **Tab Ap6: Standardized path coefficients of SEM equation 6 (PHQ-4) (R^2^ = 0.119)** | | | | |
| --- | --- | --- | --- | --- |
|  | **beta** | **s.e.** | **T** | **p** |
| Criminal victimization (CV) | 0.465 | 0.175 | 2.651 | 0.008 |
| Perceived risk of a future criminal victimization | 0.051 | 0.038 | 1.326 | 0.185 |
| Perceived ability to prevent future CV | -0.044 | 0.027 | -1.612 | 0.107 |
| Perceived safety | -0.084 | 0.039 | -2.167 | 0.030 |
| Cognitive social capital (SC) | -0.034 | 0.030 | -1.165 | 0.244 |
| Interaction term CV x SC | -0.373 | 0.164 | -2.276 | 0.023 |
| Female | 0.021 | 0.027 | 0.774 | 0.439 |
| Age | -0.009 | 0.026 | -0.353 | 0.724 |
| Education | -0.108 | 0.027 | -3.951 | 0.000 |
| Income | -0.150 | 0.036 | -4.137 | 0.000 |
| Unemployed | 0.153 | 0.070 | 2.172 | 0.030 |
| Partner | -0.022 | 0.030 | -0.730 | 0.465 |
| Intercept | 1.925 | 0.345 | 5.581 | 0.000 |
